# Preventing Airborne Transmission of SARS-CoV-2 in Hospitals and Nursing Homes

**DOI:** 10.3390/ijerph17228553

**Published:** 2020-11-18

**Authors:** Ajit Ahlawat, Sumit Kumar Mishra, John W. Birks, Francesca Costabile, Alfred Wiedensohler

**Affiliations:** 1 Leibniz Institute for Tropospheric Research, 04318 Leipzig, Germany; 2 CSIR-National Physical Laboratory, New Delhi 110012, India; sumitkumarm@gmail.com; 3 2B Technologies, 2100 Central Ave., Suite 104, Boulder, CO 80301, USA; johnb@twobtech.com; 4 Institute of Atmospheric Science and Climate (ISAC), National Research Council (CNR), I-00133 Rome, Italy; f.costabile@isac.cnr.it

The first case of the coronavirus disease 2019 (COVID-19), the novel contagious disease caused by severe acute respiratory syndrome coronavirus 2 (SARS-CoV-2), was reported in Wuhan, China in December 2019. Airborne transmission of SARS-CoV-2 has already been acknowledged by many researchers and countries around the world. There is a need to provide a proper strategy protecting the healthcare workers (HCWs) from airborne transmission of SARS-CoV-2. Additionally, it is mandatory to implement effective public health measures for emerging infectious diseases such as COVID-19 related to the airborne mode of transmission. Here, we propose some guidelines aimed at setting a benchmark for current and future viral pandemics. Firstly, we suggest the use of evaporative humidifiers along with portable air cleaners and an adequate mechanical ventilation system. The respirators without valves are recommended for HCWs during surgical and other medical procedures. Then, we recommend avoiding the use of aerosolized procedures using nebulizers and advise the use of metered-dose inhalers instead. We also recommend avoiding the use of hydrogen peroxide disinfectants for indoor cleaning. Finally, the UV-C technique should not be used too often; although known to destroy the SARS-CoV-2, it ultimately increases ozone concentrations in indoor settings.

## 1. Introduction

The novel severe acute respiratory syndrome coronavirus 2 (SARS-CoV-2), and the resulting coronavirus disease 2019 (COVID-19), was first reported in Wuhan, China, in December 2019. It has severely impacted the global economy and created a global public health crisis. The World Health Organization declared this as a global pandemic on March 11 2020. Studies have implicated the role of emitted droplets and aerosol particles in the SARS-CoV-2 transmission, particularly termed as airborne transmission [1,2]. The Canadian, Belgian and Swiss governments have acknowledged SARS-CoV-2 airborne transmission and proposed some guidelines. There are reports of aerosol particle transmission in nursing homes and hospitals [3,4,5,6,7]. Several on-field studies were carried out inside Wuhan Hospitals showing the presence of SARS-COV-2 RNA in air samples collected in the hospitals [4,5]. There are similar findings which reported the virus in air samples collected at the Nebraska University Hospital [6]. The complexity behind airborne transmission of SARS-CoV-2, especially indoors, is far from being solved, and there is the need to provide proper guidelines, to protect the healthcare workers (HCWs). Here, we address the topic in an attempt to suggest guidelines which will be helpful in controlling the on-going and future viral pandemics.

### 1.1. Airborne Transmission of SARS-CoV-2 Virus Resulting in COVID-19 Outbreak in Hospitals and Nursing Homes

The U.S. Centers for Disease Control and Prevention (CDC) has acknowledged that airborne aerosol particle transmission of SARS-CoV-2 can occur under certain specific circumstances, such as in enclosed places with inadequate ventilation [8]. This might be critical particularly within hospitals and nursing homes, where SARS-CoV-2 infection has been found to be spreading at a rapid rate despite the use of surgical masks and personal protective equipment (PPE) kits [3,4,5,6,7]. A few months previously, SARS-CoV-2 was found in aerosol particles in hospitals in Wuhan, China, farther than 2 m (6 ft) distance from emittance [4]. During another experimental study conducted at the health facilities in Wuhan, China, researchers have investigated airborne transmission of SARS-CoV-2 and the possible role of aerosol particles in SARS-CoV-2 transmission. SARS-CoV-2 viral RNA was found in 21% of the aerosol samples (n = 38) collected from Wuhan hospitals in early 2020 [5]. During the early isolation of 13 COVID-19 patients at the University of Nebraska Medical Center, air and surface samples were collected to examine viral shedding from isolated individuals. Researchers have detected viral contamination among all the samples, demonstrating that SARS-CoV-2 may be transmitted through both routes, i.e., (1) direct (droplet and person-to-person) and (2) indirect mechanisms such as from contaminated objects and airborne transmission [6]. In another case, the ventilation system of a nursing ward having a COVID-19 outbreak was investigated in addition to the routine source and contact tracing [7]. As per the report, the newly renovated ward consisted of a CO_2_-regulated energy-efficient ventilation system where the indoor air was only refreshed with outside air based on real-time CO_2_ concentration measurements, CO_2_ serving as a tracer of other components of exhaled breath such as viral particles. In this case, if the CO_2_ concentration did not exceed 1000 ppm, the ventilation cabinets recirculated indoor air back without any filtration. The low activity patients produced lower levels of CO_2_ than the set threshold standard, thereby limiting the ventilation with outside air. The recirculation of indoor air was supported by additional air conditioning units. It was suggested that this outbreak was caused by aerosol particle transmission due to inadequate ventilation. Aerosol particle transmission is implicated here, because almost all residents and Health Care Workers (HCWs) within a ward are provided with surgical masks and proper PPE kits. Among all wards, outbreaks were limited to this particular ward with a fluctuating ventilation system that recirculated unfiltered inside air. As further evidence, SARS-CoV-2 was detected on the dust filters of the air-conditioner system [7].

### 1.2. Preventive Measures Against SARS-CoV-2 Airborne Transmission in Hospitals and Nursing Homes

As a preventive measure against SARS-CoV-2 airborne transmission in enclosed spaces, indoors, and particularly in nursing homes and hospitals during cold and dry weather conditions, we advise the use of evaporative humidifiers to maintain the indoor relative humidity (RH) within the range 40–60% and reduce respiratory infection risk [9]. Indeed, evidence from laboratory experiments suggests so far that SARS-CoV-2 favors dry and cold conditions, particularly out of direct sunlight [10,11,12]. The intermediate RH range of 40–60% is considered the best option for human respiratory immunity [9]. In addition, the virus inactivation rate within aerosol particles and droplets increases at intermediate RH, i.e., 40–60%, as compared to other humidity values [13]. An optimal indoor humidity will help in reducing COVID-19 infection risks at health care facilities and other indoor buildings where ventilation systems recirculate unfiltered indoor air [14]. CO_2_ monitors should be used in parallel; however, there is a limited guarantee of protection against airborne transmission unless a safe limit for indoor CO_2_ concentration is maintained. Using evaporative humidifiers in addition to portable air cleaners and an efficient mechanical ventilation system following the American Society of Heating, Refrigerating and Air-Conditioning Engineers (ASHRAE) standards can help protect the HCWs and other hospitalized patients from being infected with SARS-CoV-2 via aerosol particle transmission. For better protection of HCWs during aerosol generating procedures and surgical procedures that produce potentially infectious aerosol particles, non-valve particle filter masks known as respirators such as N95 should be used (tightly worn without leakages). Avoid using the FFP2 and FFP3 type respirators which have an exhalation valve or vent, because these types of respirators are not sufficient for source control. Protective equipment such as eye goggles should also be worn for risk reduction [15]. Finally, in the case of patients with COVID-19, it is advised to avoid aerosol-generating procedures and treatments when possible in order to reduce the infection risk to the HCWs. The aerosol-generating treatments generally include nebulized medications through a nebulizer. Thus, to avoid the risk of aerosolization of SARS-CoV-2 through nebulization process, inhaled medications should be managed by a metered-dose inhaler when feasible, rather than using a nebulizer [15].

## 2. Concluding Remarks

To control the SARS-CoV-2 airborne transmission indoors, particularly in hospitals and nursing homes, we advise the use of evaporative humidifiers, especially in cold, dry, and low direct sunlight conditions. To ensure further protection against viral aerosol particles, use efficient portable air cleaners. An adequate mechanical ventilation system strictly adhering to ASHRAE guidelines is highly recommended. CO_2_ monitors should be used for checking CO_2_ levels (provided safe limit indoors) to protect the HCWs and other patients in the hospital rooms. Both the use of aerosolized procedures using nebulizers during medical procedures and the use of hydrogen peroxide disinfectants for indoor cleaning should be avoided. In addition, the UV-C technique should not be used too often, to avoid increases in concentration of the toxic gas ozone. HCWs must be trained properly to follow these abovementioned guidelines. They should be made aware of the risk associated with airborne transmission of SARS-CoV-2. Such guidelines, if appropriately provided by the health authorities and implemented by HCWs, could be beneficial in reducing airborne transmission risk in hospitals and nursing homes until proper and effective vaccination is developed.
